# The impact of statistical choice on variability and error in maximal respiratory mouth pressure testing

**DOI:** 10.1113/EP093680

**Published:** 2026-06-17

**Authors:** Mitch Lomax, Josh Osofa, Nicola Armstrong, Michael Tipton, Gemma Milligan

**Affiliations:** ^1^ School of Psychology, Sport and Health Sciences University of Portsmouth Portsmouth UK; ^2^ Human and Behavioural Sciences Delivery Team Defence Science Technology Laboratory Salisbury UK

**Keywords:** maximal expiratory mouth pressure, maximal inspiratory mouth pressure, measurement error, repeatability, reproducibility, respiratory mouth pressure

## Abstract

Various statistics have been used to assess repeatability and reproducibility of maximal inspiratory and expiratory mouth pressure measurements (PImax and PEmax, respectively). Few studies have examined the impact of rater experience on these values. The aims were to assess: (1) how choice of statistic impacted repeatability of PImax and PEmax; and (2) the impact of rater experience on reproducibility of PImax and PEmax. Thirty‐one healthy adult males completed one familiarisation and two experimental visits. The PImax and PEmax were assessed using a hand‐held respiratory pressure meter by three raters with different testing experience. The intraclass correlation coefficient, coefficient of variation, standard error of measurement, smallest detectable change and limits of agreement (LoA) were determined across experimental visits for a given rater and between raters to determine repeatability and reproducibility of PImax and PEmax. For PImax and PEmax, the intraclass correlation coefficient was ≥0.97 for reproducibility and 0.89–0.95 repeatability; coefficient of variation was ≤4% for reproducibility and ≤6% for repeatability; standard error of measurement was 6–9 cmH_2_O for reproducibility and 8–15 cmH_2_O for repeatability; smallest detectable change was 16–26 cmH_2_O for reproducibility and 22–40 cmH_2_O for repeatability; LoA ratio bias was −2% to 5% for reproducibility and −4% to 2% for repeatability; and LoA ratio error was 9%–17% for reproducibility and 19%–25% for repeatability. If test administrators were trained, rater experience did not impact replicate values. However, choice of statistic impacted interpretation of PImax and PEmax variability, and measurement error and reproducibility were better than repeatability.

## Abstract (highlights)

- **What is the central question of this study?**
  The effect of the choice of reproducibility and repeatability statistic on the interpretation of variation and measurement error in maximal inspiratory mouth pressure and maximal expiratory mouth pressure across separate days and between different raters with different experience has not been examined previously.
- **What is the main finding and its importance?**
  Reproducibility was unaffected by rater experience and was better than repeatability. The standard error of measurement and smallest detectable change should be used to determine the magnitude of measurement error and change required to indicate a real difference in values, whereas the limits of agreement should be used to assess agreement between different raters.

## 1 INTRODUCTION

Maximal inspiratory mouth pressure (PImax) and maximal expiratory mouth pressure (PEmax) are surrogate measures of global inspiratory and expiratory muscle strength, respectively (Gibson, 1995; Laveneziana et al., 2019). These pressures are measured during quasi‐static volitional efforts using a pressure meter attached to a mouthpiece inserted into the mouth and are tests of the neuromuscular function of respiratory musculature (Evans & Whitelaw, 2009; Laveneziana et al., 2019; Romer & McConnell, 2004).

Past studies in healthy adults show that PImax values range from ∼65 to 151 cmH_2_O, whereas PEmax values range from 89 to 237 cmH_2_O, depending on age and sex (Black & Hyatt, 1969; Bruschi et al., 1992; Ringqvist, 1966; Romer & McConnell, 2004; Silveira et al., 2021; Wilson et al., 1984). The higher PEmax values are reflective of the larger muscle mass involved with maximal expiratory versus inspiratory manoeuvres (Evans & Whitelaw, 2009). The muscles used to generate PImax and PEmax (the muscles covering the thorax, neck and abdomen) are used for multiple purposes and not only breathing. This includes postural stabilisation (Hodges et al., 1997) and coughing (LoMauro & Aliverti, 2019). The functionality of these muscles is therefore not dependent solely upon breathing requirements but is impacted by their other functions. These factors are likely to contribute to the wide range of PImax and PEmax values observed in healthy adults (Syabbalo, 1998).

Estimates of respiratory muscle strength via maximal volitional mouth pressure assessment are frequently reported in the sport and exercise performance literature (Boussana et al., 2003; Brown & Kilding, 2011; Brown et al., 2008; Johnson et al., 2007; Lomax et al., 2019; McConnell et al., 1997; Moravec et al., 2023; Verges et al., 2007; Volianitis, McConnell, Koutedakis et al., 2001). To record PImax and PEmax, a series of single maximal inspiratory and expiratory manoeuvres are made, with the highest of each being recorded for subsequent analysis (Sapienza et al., 2002). The similarity of replicate measurements of PImax and PEmax is affected by a number of factors, which include the following: the inherent variability of the equipment used; biological variability of participants; resting state of the respiratory muscles; lung volumes from which PImax and PEmax are obtained; ability of participants to coordinate the required muscle action; motivation and capability of participants to produce maximal effort; stability of the environment in which assessments are made, including equipment and mouthpiece choice; familiarity of participants with the manoeuvres; and, possibly, some characteristics of the investigator administering the assessments, such as experience (Bartlett & Frost, 2008; Bruschi et al., 1992; de Vet et al., 2006; Gibson, 1995; McConnell, 2016; Syabbalo, 1998). These factors all contribute to variability and measurement error, which is how much the measured value differs from the true value (Bartlett & Frost, 2008) and consists of both systematic error (bias) and random error (imprecision) (Atkinson & Nevill, 1998; Weir, 2005). It is therefore important to standardise as much as possible the conditions in which replicate PImax and PEmax measurements are made to reduce measurement error.

There is no gold standard method used for assessing the repeatability (variation in repeat measurements in identical conditions) or reproducibility (degree to which repeat measurements in participants provide similar results in changing conditions) of replicate outcome measures (Bartlett & Frost, 2008; de Vet et al., 2006). Instead, a range of approaches is used depending upon the aspect of measurement error or variability of interest, whether the data are homo‐ or heteroscedastic and are normally distributed. The methods include the coefficient of variation (CV), intraclass correlation coefficient (ICC), standard error of measurement (SEM), smallest detectable change (SDC; also known as the smallest real difference and repeatability coefficient) and the limits of agreement (LoA), all of which convey different information (Atkinson & Nevill, 1998; Bartlett & Frost, 2008; de Vet et al., 2006; Eilasziw et al., 1994; Stratford & Goldsmith, 1997; Vaz et al., 2013; Weir, 2005).

In the sport and exercise science literature, those studies that have attempted to evaluate the variation or measurement error in replicate PImax and PEmax measurements have reported the CV (Gonzales & Scheuermann, 2006; Lomax et al., 2017; Volianitis et al., 1999, 2001), ICC (Brown & Kilding, 2011; Lomax & McConnell, 2009; Lomax et al., 2014), SDC (Maillard et al., 1998; Volianitis et al., 1999, 2001) and LoA (Lomax & McConnell, 2009; Romer & McConnell, 2004). Others have adopted a simpler approach, deeming the variation to be acceptable when the highest of multiple measures (usually three) are within 5% (Brown et al., 2008; Johnson et al., 2007; Verges et al., 2007) or 10% (Boussana et al., 2003; Lomax et al., 2019; Moravec et al., 2023; Santos et al., 2011) of each other.

No studies have examined how the choice of repeatability and reproducibility statistics impacts the variation and measurement error in replicate PImax and PEmax measurements in healthy adults. This is important because it might influence the magnitude of change required to indicate a real change in PImax or PEmax (e.g., in response to a training intervention) versus a change attributable to measurement error (e.g., inherent variation in equipment or different test administrators).

The aims of the present study were: (1) to assess how the choice of repeatability statistic impacted replicate PImax and PEmax values and subsequent interpretation of day‐to‐day variability or measurement error; and (2) to assess the impact of test administrators (raters) with different degrees of experience on the reproducibility of PImax and PEmax. It was hypothesised that the choice of repeatability statistic would not affect the interpretation of replicate PImax and PEmax values, nor the estimation of day‐to‐day variability or measurement error. Likewise, it was hypothesised that reproducibility of PImax and PEmax would not differ between raters with different levels of experience.

## 2 MATERIALS AND METHODS

### 2.1 Ethical approval

The study was conducted in accordance with the latest version of the *Declaration of Helsinki*, except for registration in a database, and approved by the UK Ministry of Defence Research Ethics Committee (protocol code 1065/MODREC/20 on 11 November 2020). All participants provided written informed consent before participating in the study.

### 2.2 Participants

A convenience sample of 32 healthy, active males volunteered for this study. This consisted of serving infantry soldiers and active healthy males. Participants were free from symptoms of coronavirus 2019, aged 18–50 years, met the UK government recommended weekly physical activity level of 150 min of moderate‐intensity exercise or 75 min of vigorous exercise, had a resting blood pressure between 90 and 140 mmHg and between 60 and 90 mmHg for systolic and diastolic pressures, respectively (blood pressure ranges permitted in our laboratory) and were free from diagnosed cardiovascular, metabolic or respiratory conditions, including asthma. Females were excluded, because it would not have been possible to recruit an equal number of male and female infantry soldiers, making sex comparisons untenable. One participant was withdrawn owing to an inability to perform technically correct PImax and PEmax manoeuvres following familiarisation. Data reported are therefore based on 31 participants.

### 2.3 Protocol

Participants were assessed on three separate occasions (visits) in an indoor laboratory. Each visit was separated by 24 h of recovery, during which exercise training was avoided, and occurred at approximately the same time of day to minimise diurnal variation. The mean and SD for barometric pressure, temperature and humidity were 764.4 ± 4.4 mmHg, 20.2°C ± 1.6°C and 49% ± 7%, respectively.

The first visit served as PImax and PEmax familiarisation, with visits 2 and 3 used for experimental data collection. Participants were deemed proficient at PImax and PEmax manoeuvres during familiarisation when the highest of three technically proficient manoeuvres were within 10 cmH_2_O or 10% of one another (Laveneziana et al., 2019; McConnell, 2016). On visits 2 and 3, PImax and PEmax of participants were assessed by three separate raters. The three raters varied in PImax and PEmax assessment experience. Rater 1 (Rater_1_) had ∼1 year of PImax and PEmax testing experience, rater 2 (Rater_2_) had >15 years of PImax and PEmax testing experience, and rater 3 (Rater_3_) had 1 month of PImax and PEmax testing experience and was naive to these assessments before the present study. The competency (in line with the instructions below) of raters 1 and 3 was confirmed by Rater_2_ before testing began.

All raters were given the same pre‐study PImax and PEmax training, which included watching a bespoke in‐house PImax and PEmax instructional digital recording. This recording included the following instructions along with a demonstration: each PImax manoeuvre must be initiated from residual volume and whilst standing; each PEmax manoeuvre must be initiated from total lung capacity and whilst standing; a nose clip must be worn; each PImax and PEmax effort must be maximal in nature; each maximal effort must be sustained for at least 2–3 s; and a minimum of 30 s rest must be given between repeated manoeuvres (Laveneziana et al., 2019; McConnell, 2016). Raters were not required to follow a pre‐set instructional script but were instead permitted to use their own words when instructing participants. Examples included ‘slowly empty your lungs so that nothing is left’ and ‘slowly squeeze all of the air out of your lungs’ for residual volume; ‘totally fill up your lungs with air’ and ‘take a big breath in and breathe in until you can't get any more air in’ for total lung capacity; and ‘hold, hold, hold’ and counting ‘1, 2, 3’ for sustaining maximal effort.

Participants used the same respiratory mouth pressure meter (RPM, Micro Medical, UK) throughout, which was connected to a flanged rubber mouthpiece. Participants undertook all PImax and PEmax manoeuvres in a standing position while wearing a nose clip. The PImax was assessed first, and the PImax and PEmax values recorded represented the maximum average pressure sustained over 1 s during a given 2–3 s manoeuvre.

During visits 2 and 3, participants performed nine technically proficient PImax manoeuvres followed by nine technically proficient PEmax manoeuvres. Each rater was responsible for administering three proficient PImax and three proficient PEmax manoeuvres, and to do so separately from each other rater. If a rater deemed an effort to be technically incorrect or lacking in effort, the participant was required to repeat that manoeuvre. Raters were blinded to each other's PImax and PEmax values. The order in which raters administered their PImax and PEmax manoeuvres was randomised using a freely available on‐line random sequence generator (random.org).

### 2.4 Statistical analyses

#### 2.4.1 Approach overview

Given that PImax and PEmax are typically reported as the highest value reported in a series of measurements (McConnell, 2016), the highest value per rater and per visit were used in all calculations.

Repeatability and reproducibility were compared between two visits (Lomax & McConnell, 2009; Romer & McConnell, 2004; Silveira et al., 2021; Volianitis et al., 2001; Windisch et al., 2004). For repeatability, PImax and PEmax were compared between visits 2 and 3 for a given rater; and to assess reproducibility, PImax and PEmax values recorded by each rater within visits 2 and 3 were compared.

Alpha was set at ≤0.05, and data are reported as the mean and SD unless otherwise stated. IBM SPSS (v.28) and Microsoft Excel were used for analyses. Leven's test of homogeneity of variance indicated that PImax and PEmax were homoscedastic. The Shapiro–Wilks test of normality indicated a mixture of normal and non‐normal distribution (significant deviation from normality was observed in PImax at visit 2 and in PEmax at visit 3 for Rater_1_ and at visit 3 for Rater_1_ and Rater_2_). We chose not to logarithmically transform all the data owing to the lack of heteroscedasticity and to allow data to be interpreted with regard to absolute differences in the original unit of measurement. However, we acknowledge that data could have been log‐transformed, and the LoA were log‐transformed for reasons outlined below.

#### 2.4.2 Difference testing

Because there is no non‐parametric version of a two‐way repeated‐measures ANOVA, significant differences between raters and visits 2 and 3 for PImax and PEmax were assessed using a two‐way (visit × rater) repeated‐measures ANOVA. Given that significant differences were observed in PEmax between raters, parametric one‐way repeated‐measures ANOVAs with Bonferroni correction were used to identify differences between raters for visit 2 and Wilcoxon signed‐rank tests (Rater_1_) and Student's paired *t*‐tests (Rater_2_, Rater_3_) with Bonferroni correction were used to identify differences between raters for visit three. Additionally, given that an interaction effect was observed in PEmax, Wilcoxon signed‐rank tests (Rater_1_) and Student's paired *t*‐tests (Rater_2_, Rater_3_) were used to ascertain where the interaction lay. When differences were observed, parametric data effect sizes were calculated using Cohen's *d*, with 0.2 deemed small, 0.6 moderate, 1.2 large, 2.0 very large and 4.0 extremely large (Hopkins et al., 2009). For non‐parametric data, *r* was used, where *r* is the *z*‐score divided by the square root of the total number of observations. A value of 0.1 is deemed small, 0.3 medium and ≥0.5 large (Fritz & Morris, 2012).

#### 2.4.3 CV

Repeatability of values between visits 2 and 3 for Rater_1_ (CV_1_), Rater_2_ (CV_2_) and Rater_3_ (CV_3_) and the reproducibility of values between the three raters for visit 2 (CV_1–3, visit 2_) and visit 3 (CV_1–3_, _visit 3_) were calculated in accordance with Equation (1) (Atkinson & Nevill, 1998):

(1) $$\mathrm{CV}=\left(\mathrm{SD}/\mathrm{mean}\right)\times 100$$

There are no fixed criteria for interpreting the CV. Nevertheless, the smaller the value, the better.

#### 2.4.4 ICC

Repeatability of values between visits 2 and 3 for Rater_1_ (ICC_1_), Rater_2_ (ICC_2_) and Rater_3_ (ICC_3_) and the reproducibility of values between the three raters for visit 2 (ICC_1–3, visit 2_) and visit 3 (ICC_1–3, visit 3_) were calculated in accordance with Equation (2), with the random error and bias included in the calculation (Vincent, 2005):

(2) $$\mathrm{ICC}=\left(\mathrm{MSR}-\mathrm{MSC}+E\right)/\mathrm{MSR}$$

where MSC + *E* = (SSC + SSE)/(dfc + dfe).

A single‐factor (one‐way), within‐subjects ANOVA (repeated‐measures) was used to ascertain the above values. MSR is the mean square error variability among participants, which was derived from the tests of between‐subject effects. MSC + *E* is the sum of the changes in the mean and error, SSC is the sum of squares for the treatment (i.e., rater), SSE is the sum of squares for error, dfc is the degrees of freedom for the treatment, and dfe is the degrees of freedom for error.

Correlation thresholds are used to interpret ICCs. An ICC value of 0.00 indicates that all variability is attributable to measurement error, whereas a value of 1.00 indicates the absence of measurement error (Bartlett & Frost, 2008). Within this range, ≥0.90 is considered high (i.e., low measurement error), 0.80–0.89 moderate, and <0.80 is considered questionable (Atkinson & Nevill, 1998; Vincent, 2005). A value of ≥0.80 is deemed acceptable (Eliasziw et al., 1994).

#### 2.4.5 SEM and the SDC

Repeatability of values between visits 2 and 3 for Rater_1_ (SEM_1_), Rater_2_ (SEM_2_) and Rater_3_ (SEM_3_) and the reproducibility of values between the three raters for visit 2 (SEM_1–3, visit 2_) and visit 3 (SEM_1–3, visit 3_) were calculated in accordance with Equation (3) (Vincent, 2005) and the 95% confidence interval (CI) in accordance with Equation (4) (Stratford & Goldsmith, 1997):

(3) $$\mathrm{SEM}=\sqrt{\mathrm{MSE}}\;$$

where MSE is the mean square error determined by a single‐factor, within‐subjects ANOVA.

(4) $$\left[\sqrt{\frac{\mathrm{SSE}}{\chi^{2}\alpha ,\mathrm{dfe}},\sqrt{\frac{\mathrm{SSE}}{\chi^{2}1-\alpha ,\mathrm{dfe}}}}\right]$$

where SSE is the sample error variance derived from ANOVA, χ^2^α is the χ^2^ value for alpha (one‐tailed), and dfe is the degrees of freedom for error associated with SSE.

The SEM was then used to determine the SDC in accordance with Equation (5) (de Vet et al., 2006):

(5) $$\mathrm{SDC}=1.96\times \sqrt{\;2}\times \mathrm{SEM}$$

where 1.96 is the 95% CI *z*‐score.

There are no fixed criteria for interpreting the SEM or SDC. Nevertheless, the smaller the values the better.

#### 2.4.6 LoA

Repeatability of values between visits two and three for Rater_1_ (LoA_1_), Rater_2_ (LoA_2_) and Rater_3_ (LoA_3_) was calculated as visit 2 minus visit 3. Reproducibility between raters was determined by comparing each rater with each other rater per visit, with reference to the rater with the greater experience. Thus, Rater_1_ was compared with Rater_2_ (LoA_1,2 visit 2_, LoA_1,2 visit 3_, calculated as Rater_2_ minus Rater_1_) and Rater_3_ (LoA_1,3 visit 2_, LoA_1,3 visit 3_, calculated as Rater_1_ minus Rater_3_), and Rater_2_ was compared with Rater_3_ (LoA_2,3 visit 2_, LoA_2,3 visit 3_, calculated as Rater_2_ minus Rater_3_). These were determined in accordance with Equation (6) (Bland & Altman, 1986):

(6) $$\mathrm{LoA}=\mathrm{mean}\;\mathrm{difference}\pm \left(1.96\times \mathrm{SD}\;\mathrm{of}\;\mathrm{the}\;\mathrm{difference}\right)$$

The precision of the LoA were subsequently calculated in accordance with Equation (7) (Bland & Altman, 1986):

(7) $$\begin{array}{c} 95\%\mathrm{CI}\;\mathrm{for}\;\mathrm{bias}=\mathrm{mean}\;\mathrm{difference}\pm \left(t\times \mathrm{SE}\;\mathrm{of}\;\mathrm{the}\;\mathrm{difference}\right)\\ 95\%\mathrm{CI}\;\mathrm{for}\;\mathrm{lower}\;\mathrm{LoA}=\mathrm{lower}\;\mathrm{LoA}\pm \left(t\times \mathrm{SE}\;\mathrm{of}\;\mathrm{the}\;\mathrm{LoA}\right)\\ 95\%\mathrm{CI}\;\mathrm{for}\;\mathrm{upper}\;\mathrm{LoA}=\mathrm{upper}\;\mathrm{LoA}\pm \left(t\times \mathrm{SE}\;\mathrm{of}\;\mathrm{the}\;\mathrm{LoA}\right) \end{array}$$

where *t* is the *t*‐test statistic based on the 95% CI (two‐tailed) and degrees of freedom of *n* − 1, and SE is the standard error.

In the case of LoA, assumptions of normality or homogeneity were based on the difference in values between any given two raters or two visits rather than the absolute values observed per rater; the latter was how normality and homogeneity were examined prior to ICC, CV and SEM/SDC assessments. Given that assumptions of normality or homogeneity were violated in some instances, data were logarithmically transformed using the natural logarithm, anti‐logged and displayed as ratios (Bartlett & Frost, 2008; Bland & Altman, 1986; Lomax et al., 2019; Romer & McConnell, 2004). To aid with interpretation, the LoA ratio data were also expressed as percentage data.

## 3 RESULTS

The mean and SD for age, mass and height were 22 ± 5 years, 79.9 ± 11.3 kg and 1.79 ± 0.07 m, respectively. No participant had any previous experience of PImax and PEmax assessments.

### 3.1 Maximal inspiratory and expiratory mouth pressure values

The absolute (in centimetres of water) group mean and SD PImax and PEmax values per rater and per visit are displayed in Table 1. There were no differences between raters (*F*_2,60_ = 2.489, *P* = 0.092, power = 0.481) or visits (*F*_1,30_ = 0.119, *P* = 0.731, power = 0.063) for PImax. There was no difference between visits (*F*_1,30 _= 0.139, *P* = 0.712, power = 0.065) for PEmax, but differences were observed between raters (*F*_2,60 _= 7.184, *P* = 0.004, power = 0.865, *d* = 0.15 to −0.24, *r* = −0.45), and an interaction was also observed (*F*_2,60_ = 6.188, *P* = 0.007, power = 0.816).

**TABLE 1:** Maximal inspiratory and expiratory mouth pressures per rater and per visit: Group mean and SD (*n* = 31).

Comparison	Rater_1_	Rater_2_	Rater_3_
PImax, visit 2, cmH_2_O	133 ± 24	132 ± 25	131 ± 22
PImax, visit 3, cmH_2_O	133 ± 28	134 ± 24	130 ± 24
PEmax, visit 2, cmH_2_O	179 ± 33^b^	187 ± 33	182 ± 33^b^
PEmax, visit 3, cmH_2_O	187 ± 36^a^, ^c^	186 ± 34	179 ± 34^b^

*Note*: PImax: maximal inspiratory mouth pressure. PEmax: maximal expiratory mouth pressure. The respiratory muscle strength of the group as a whole exceeded the age‐predicted normative values for PImax (99–123 cmH_2_O) and PEmax (142–164 cmH_2_O) expected in British adult males (Wilson et al., 1984).

^a^ *P* ≤ 0.05 different from visit 2.

^b^ *P* ≤ 0.01 different from Rater_2_.

^c^ *P* ≤ 0.01 different from Rater_3_.

### 3.2 CV

As can be seen in Table 2, the CV was no greater than 4% for reproducibility and 6% for repeatability of PImax and PEmax.

**TABLE 2:** Coefficient of variation for maximal inspiratory and expiratory mouth pressures and the expected variation in pressure values between raters and within raters (*n* = 31).

Comparison	PImax CV	PImax variation		PEmax CV	PEmax variation	
	(%)	Highest (cmH_2_O)	Lowest (cmH_2_O)	(%)	Highest (cmH_2_O)	Lowest (cmH_2_O)
CV_1–3 visit 2_	4	8 (203 × 0.04)	4 (91 × 0.04)	4	10 (260 × 0.04)	4 (112 × 0.04)
CV_1–3 visit 3_	2	4 (206 × 0.02)	2 (89 × 0.02)	4	5 (253 × 0.02)	2 (105 × 0.02)
CV_1_	6	12 (206 × 0.06)	5 (89 × 0.06)	6	16 (260 × 0.06)	6 (105 × 0.06)
CV_2_	5	10 (203 × 0.05)	4 (89 × 0.05)	5	13 (253 × 0.05)	6 (111 × 0.05)
CV_3_	5	10 (197 × 0.05)	4 (89 × 0.05)	6	12 (247 × 0.05)	5 (106 × 0.05)

*Note*: CV: coefficient of variation. PImax: maximal inspiratory mouth pressure. PEmax: maximal expiratory mouth pressure. Variations are calculated based on the highest and lowest PImax and PEmax values observed for a given comparison. The calculations for variation are stated in parentheses and represent the highest or lowest PImax/PEmax participant value observed multiplied by the CV.

### 3.3 ICC

The reproducibility and repeatability for PImax was high (Table 3). Reproducibility between raters was also high for PEmax, as was repeatability for Rater_2_, but it was only moderate for Rater_1_ and Rater_3_ (Table 3). Nevertheless, all ICCs were deemed acceptable (>0.80).

**TABLE 3:** Intraclass correlation coefficient and interpretations of reproducibility and repeatability (*n* = 31).

Comparison	PImax		PEmax	
	ICC	Interpretation	ICC	Interpretation
ICC_1–3 visit 2_	0.98	Highly reproducible	0.97	Highly reproducible
ICC_1–3 visit 3_	0.98	Highly reproducible	0.97	Highly reproducible
ICC_1_	0.93	Highly repeatable	0.89	Moderately repeatable
ICC_2_	0.95	Highly repeatable	0.93	Highly repeatable
ICC_3_	0.93	Highly repeatable	0.89	Moderately repeatable

### 3.4 SEM and SDC

The SEM and SDC for PImax and PEmax are displayed in Table 4. Both were smaller for PImax compared with PEmax. Additionally, the repeatability SEM and SDC were worse than the reproducibility statistics, indicating that between‐day variability for a given rater was larger than the same‐day variability of different raters.

**TABLE 4:** Standard error of measurement and smallest detectable change for maximal inspiratory and expiratory mouth pressures between raters and within raters (*n* = 31).

Comparison	PImax			PEmax		
	SEM (cmH_2_O)	95% CI SEM (cmH_2_O)	SDC (cmH_2_O)	SEM (cmH_2_O)	95% CI SEM (cmH_2_O)	SDC (cmH_2_O)
SEM_1–3 visit 2_	6	5–8	18	9	7–11	24
SEM_1–3 visit 3_	6	5–7	16	9	8–11	26
SEM_1_	10	8–13	27	15	12–19	40
SEM_2_	8	6–11	22	12	10–16	34
SEM_3_	8	7–11	23	15	12–20	41

### 3.5 LoA

**FIGURE 1:**
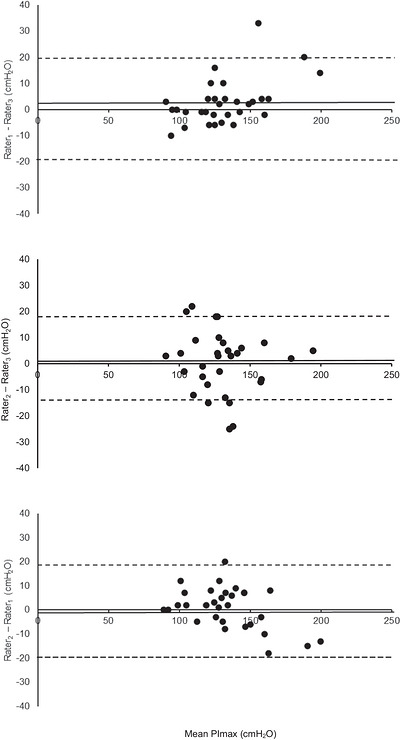
Bland–Altman plots for the reproducibility of PImax: visit 2 (*n* = 31). Note that the bias (continuous line) and 95% limits of agreement (dashed lines) are also shown.

**FIGURE 2:**
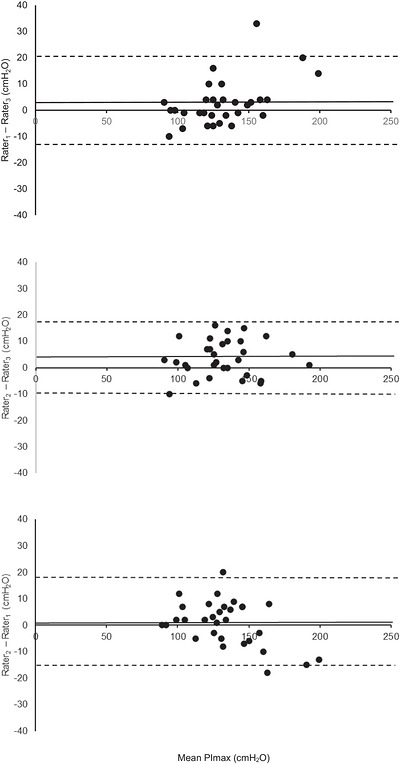
Bland–Altman plots for the reproducibility of PImax: visit 3 (*n* = 31). Note that the bias (continuous line) and 95% limits of agreement (dashed lines) are also shown.

**FIGURE 3:**
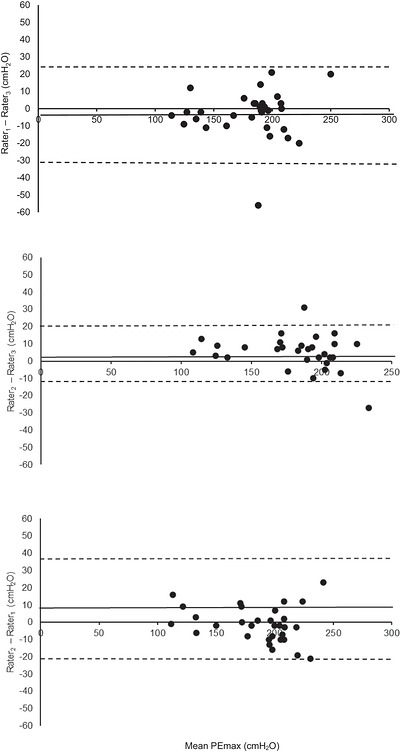
Bland–Altman plots for the reproducibility of PEmax: visit 2 (*n* = 31). Note that the bias (continuous line) and 95% limits of agreement (dashed lines) are also shown.

**FIGURE 4:**
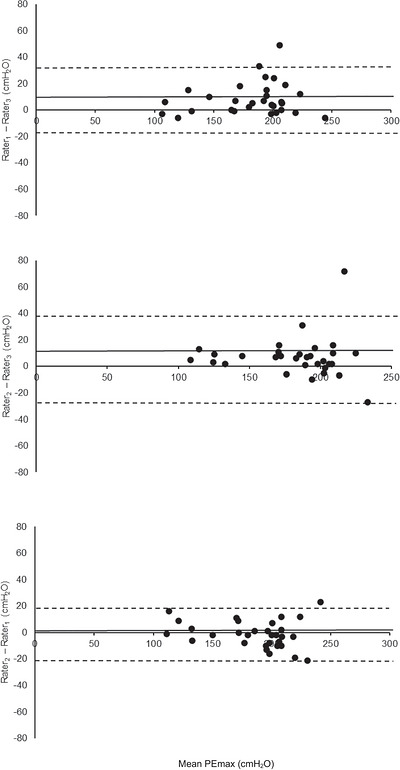
Bland–Altman plots for the reproducibility of PEmax: visit 3 (*n* = 31). Note that the bias (continuous line) and 95% limits of agreement (dashed lines) are also shown.

**FIGURE 5:**
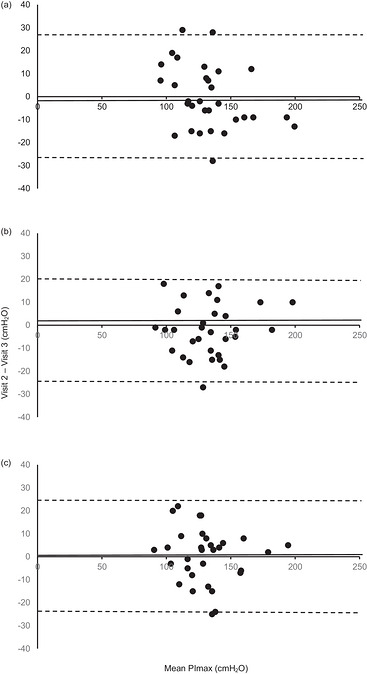
Bland–Altman plots for the repeatability of PImax between visits 2 and 3 per rater (*n* = 31). (a) Rater_1_. (b) Rater_2_. (c) Rater_3_. The bias (continuous line) and 95% limits of agreement (dashed lines) are also shown.

**FIGURE 6:**
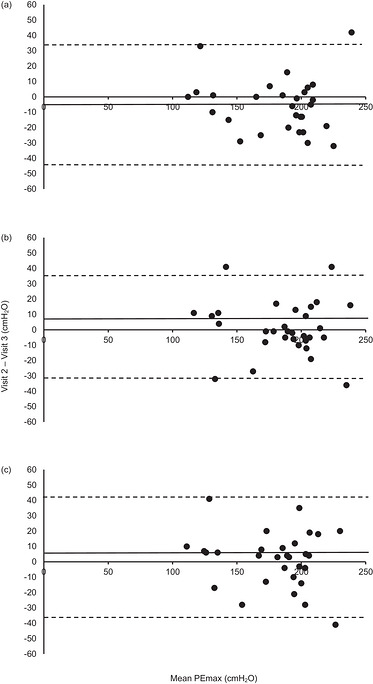
Bland–Altman plots for the repeatability of PEmax between visits 2 and 3 per rater (*n* = 31). (a) Rater_1_. (b) Rater_2_. (c) Rater_3_. The bias (continuous line) and 95% limits of agreement (dashed lines) are also shown.

Significant bias was observed between raters, with bias being more evident for PEmax than for PImax. In the case of PImax, it was observed only between Rater_2_ and Rater_3_ for visit 3 (*P* = 0.006). For PEmax it was observed between Rater_2_ and Rater_1_ for visit 2 (*P* = 0.008), between Rater_1_ and Rater_3_ for visit 3 (*P* = 0.001) and between Rater_2_ and Rater_3_ for both visit 2 (*P* = 0.003) and visit 3 (*P* = 0.017). For repeatability there was no significant bias observed in PImax between visits for any rater (Rater_1_, *P* = 0.908; Rater_2_, *P* = 0.284; Rater_3_, *P* = 0.891), but there was significant bias observed (*P* = 0.044) in PEmax for Rater_1_ (Rater_2_, *P* = 0.781; Rater_3_, *P* = 0.338). Thus, bias was more evident between raters than for a given rater between testing days. Additionally, random error was larger than bias in all comparisons (Tables 5 and 6). Bland–Altman plots for PImax and PEmax are shown in Figures 1, 2, 3, 4, 5, 6.

**TABLE 5:** Ratio limits of agreement for maximal inspiratory mouth pressure, including estimated precision of the limits of agreement between raters and within rater (*n* = 31).

Comparison	95% LoA		Bias			Random error			
	Lower	Upper	Ratio	SE	95% CI lower‐upper	Ratio	SE	95% CI lower LoA	95% CI Upper LoA
LoA_1,2 visit 2_	0.85 (15)	1.15 (15)	0.99 (−1)	0.01	−0.04 to 0.02	1.16 (16)	0.02	0.80–0.90	1.10–1.20
LoA_1,3 visit 2_	0.90 (10)	1.15 (15)	1.01 (1)	0.01	−0.01 to 0.04	1.13 (13)	0.02	0.86–0.94	1.11–1.19
LoA_2,3 visit 2_	0.88 (12)	1.15 (15)	1.00 (0)	0.01	−0.02 to 0.03	1.14 (14)	0.02	0.84–0.92	1.10–1.19
LoA_1,2 visit 3_	0.90 (10)	1.14 (14)	1.01 (1)	0.01	−0.01 to 0.03	1.13 (13)	0.02	0.86–0.94	1.10–1.18
LoA_1,3 visit 3_	0.90 (10)	1.15 (15)	1.02 (2)	0.01	−0.01 to 0.04	1.13 (13)	0.02	0.86–0.94	1.11–1.19
LoA_2,3 visit 3_	0.92 (8)	1.14 (14)	1.03^a^ (3)	0.01	0.01 to 0.05	1.11 (11)	0.02	0.89–0.96	1.11–1.18
LoA_1_	0.81 (19)	1.25 (25)	1.00 (0)	0.02	−0.04 to 0.05	1.24 (24)	0.03	0.74–0.88	1.18–1.32
LoA_2_	0.83 (17)	1.17 (17)	0.98 (−2)	0.02	−0.05 to 0.02	1.19 (19)	0.03	0.77–0.88	1.11–1.23
LoA_3_	0.84 (16)	1.21 (21)	1.01 (1)	0.02	−0.03 to 0.04	1.20 (20)	0.03	0.77–0.90	1.15–1.27

*Note*: CI: confidence interval. LoA: limits of agreement. SE: standard error. To help with interpretation of the LoA, bias and random error, the values in parentheses reflect expression as a percentage.

^a^ *P* ≤ 0.01 bias observed.

**TABLE 6:** Ratio limits of agreement for maximal expiratory mouth pressure, including estimated precision of the limits of agreement between raters and within rater (*n* = 31).

Comparison	95% LoA		Bias			Random error			
	Lower	Upper	Ratio	SE	95% CI lower‐upper	Ratio	SE	95% CI lower LoA	95% CI Upper LoA
LoA_1,2 visit 2_	0.89 (11)	1.23 (23)	1.04^b^ (4)	0.01	0.01 to 0.07	1.17 (17)	0.03	0.84–0.94	1.17–1.28
LoA_1,3 visit 2_	0.85 (15)	1.13 (13)	0.98 (−2)	0.01	−0.05 to 0.01	1.15 (15)	0.02	0.80–0.90	1.09–1.18
LoA_2,3 visit 2_	0.94 (6)	1.11 (11)	1.02^b^ (2)	0.01	0.01 to 0.04	1.09 (9)	0.01	0.92–0.97	1.09–1.14
LoA_1,2 visit 3_	0.89 (11)	1.11 (11)	1.00 (0)	0.01	−0.02 to 0.02	1.11 (11)	0.02	0.86–0.93	1.08–1.15
LoA_1,3 visit 3_	0.92 (8)	1.18 (18)	1.05^b^ (5)	0.01	0.02 to 0.07	1.13 (13)	0.02	0.88–0.96	1.14–1.23
LoA_2,3 visit 3_	0.90 (10)	1.21 (21)	1.04^a^ (4)	0.01	0.01 to 0.07	1.16 (16)	0.02	0.85–0.95	1.16–1.26
LoA_1_	0.77 (23)	1.20 (20)	0.96^a^ (−4)	0.02	−0.08 to 0.002	1.25 (25)	0.04	0.70–0.84	1.13–1.27
LoA_2_	0.83 (17)	1.23 (23)	1.01 (1)	0.02	−0.03 to 0.04	1.22 (22)	0.03	0.76–0.89	1.16–1.29
LoA_3_	0.82 (18)	1.28 (28)	1.02 (2)	0.02	−0.02 to 0.07	1.25 (25)	0.04	0.74–0.89	1.21–1.36

*Note*: CI: confidence interval. LoA: limits of agreement. SE: standard error. To help with interpretation of the LoA, bias and random error, the values in parentheses reflect expression as a percentage.

^a^ *P* ≤ 0.05 and

^b^ *P* ≤ 0.01 bias observed.

### 3.6 Data deposition

See data availability statement in Additional information.

## 4 DISCUSSION

The novel aims of the present study were to assess how the choice of statistic impacted repeatability of PImax and PEmax and to assess the impact of different raters with various degrees of experience on the reproducibility of PImax and PEmax in a sample of young healthy adult males. The key recommendations/findings are as follows: (1) the SEM and SDC should be used for assessing measurement error and the change required to indicate a ‘real’ difference in PImax and PEmax values; (2) the LoA should be used for assessing agreement in PImax and PEmax between raters; (3) the ICC should be used to assess relative consistency in PImax and PEmax between participants; (4) the CV should be used to assess the spread of PImax and PEmax values around the mean; (5) reproducibility of PImax and PEmax was better than repeatability; and 6) rater experience had little impact on the reproducibility of PImax and PEmax.

Combinations of ICC, CV, SEM, SDC and LoA are routinely adopted in papers assessing the repeatability or reproducibility of PImax and PEmax in healthy adults. However, these statistics do not convey the same information. Additionally, both the ICC and CV are dimensionless statistics, meaning that values are not expressed in the same unit as the measured value. In contrast, the SEM, SDC and LoA are reported in the same units of measurement (Atkinson & Nevill, 1998; Eliasziw et al., 1994; Weir, 2005).

Studies on healthy adults have reported ICC values of 0.88–0.99 for PImax (Lomax & McConnell, 2009; Lomax et al., 2012; Maillard et al., 1998; Silveira et al., 2021) and 0.78–0.97 for PEmax (Lomax et al., 2012; Silveira et al., 2021). The results of the present study are consistent with these observations. Regardless of rater, PImax was highly repeatable, with unexplained error (1 − ICC) accounting for only 5%–7% of its value, while PEmax was moderately‐to‐highly repeatable, with unexplained error accounting for 7%–11% of its value (Table 3).

The ICC cannot be used directly to estimate future PImax and PEmax values. Instead, it must be used indirectly, such as in the calculation of the SEM (Atkinson & Nevill, 1998; Vincent, 2005; Weir, 2005). The ICC is also reflective of the ability of a test to differentiate between values of different individuals, hence it does not assess test–retest variability. It is therefore a test of relative reliability, not absolute reliability. Consequently, high values can mask poor day‐to‐day repeatability if between‐subject variability (i.e., heterogeneity) is high (Quan & Shih, 1996; Weir, 2005). For these reasons, it is not advisable to use the ICC in isolation if an index of test–retest error is also needed (Atkinson & Nevill, 1998; Weir, 2005).

Unlike the ICC, the CV is a measure of absolute reliability and is sensitive to a shift in the scale of measurement (Atkinson & Nevill, 1998; Quan & Shih, 1996). In the present study, the repeatability CV for both PImax and PEmax was 5%–6% across raters (Table 2). This is consistent with the wider literature in healthy individuals, where a CV of 5%–10% has been reported for both PImax (Aldrich & Spiro, 1995; Black & Hyatt, 1969; Lomax et al., 2015; Maillard et al., 1998; Reiter et al., 2006; Ringqvist, 1966; Volianitis et al., 1999; Volianitis, McConnell, & Jones, 2001) and PEmax (Black & Hyatt, 1969; Lomax et al., 2015; Man et al., 2003; Ringqvist, 1966; Wilson et al., 1984), although a value of <2% for PImax has been observed when data are logarithmically transformed (Lomax & McConnell, 2009), and a CV as high as 25% has reported in older females (Reiter et al., 2006).

Whether or not the CV is deemed acceptable should be based on the analytical goal in question and not an arbitrary threshold. If the CV is greater than the expected change, the CV is too high (Atkinson & Nevill, 1998). As an example, the CV for Rater_2_ was 5% for both PImax and PEmax (Table 2). This means that the participants with the lowest PImax (89 cmH_2_O) and PEmax (111 cmH_2_O) recorded by Rater_2_ could produce values that vary by 4 cmH_2_O for PImax and by 6 cmH_2_O for PEmax, whereas those with the highest values (PImax, 203 cmH_2_O; PEmax, 253 cmH_2_O) could produce values that vary by as much as 10 cmH_2_O for PImax and 13 cmH_2_O for PEmax. This means that any hypothesised change must exceed these values for the CV to be considered acceptable. Importantly however, the CV is not used to predict future values because it is not a direct measure of precision. Instead, it describes the spread of values around the mean and can also be used to compare the level of variation between different assessment tools (Atkinson & Nevill, 1998). In contrast, the SEM is affected by error variation only and is therefore ideally suited for predicting future PImax and PEmax values (Stratford & Goldsmith, 1997). The SDC can then identify the value needed to indicate that a ‘real’ change has occurred (Weir, 2005).

Continuing with the example of Rater_2_, the SEM was 8 cmH_2_O for PImax and 12 cmH_2_O for PEmax (Table 4). It is likely (95% level of confidence) that the individuals with the lowest PImax and PEmax values would have a true value that falls within 81–97 cmH_2_O for PImax and 99–123 cmH_2_O for PEmax. With a corresponding SDC of 22 cmH_2_O for PImax and 34 cmH_2_O for PEmax (Table 4), a change of >22 cmH_2_O in PImax and >34 cmH_2_O in PEmax would be required to be confident (95% level of confidence) that a real difference was being observed. Changes of any less could simply reflect measurement error. It should also be remembered that accuracy of the measurement equipment will be an inherent part of variability, and the accuracy of the device used in the present study was ±3% according to manufacture specifications. This magnitude of SDC is consistent with that reported for PImax (25–31 cmH_2_O) in healthy adults (Maillard et al., 1998; Volianitis et al., 1999; Volianitis, McConnell, & Jones, 2001). However, such a magnitude of change could be too great to mask subtler changes, particularly reductions in respiratory muscle strength associated with disease progression.

As an example, in patients with chronic obstructive pulmonary disease (COPD), a mean difference of only 19 cmH_2_O in PImax has been observed in patients categorised with mild, moderate or severe COPD (Terzano et al., 2008). These different COPD stages require different levels of medical intervention, and the measurement of respiratory muscle strength might help in monitoring COPD progression (Terzano et al., 2008) but not if the SDC exceeds the predicted change. In neuromuscular or autoimmune diseases associated with respiratory muscle weakness, smaller but clinically significant changes might be observed. Routine measurement of respiratory muscle strength is therefore an important part of monitoring disease progression and treatment efficacy (Goswami et al., 2002; Keenan et al., 1995; Khan et al., 2023). For example, Keenan et al. (1995) found that in patients with generalised myasthenia gravis (a neuromuscular disease), respiratory muscle weakness was evident, and dyspnoea was greater than in non‐suffers. PImax was on average 67 cmH_2_O (70% of predicted), which is indicative of clinical inspiratory muscle weakness. However, 20 min after the administration of neostigmine (an acetylcholinesterase inhibitor), PImax increased to 80 cmH_2_O (83% of predicted). Given that a PImax of 80 cmH_2_O generally excludes clinically important inspiratory muscle weakness (Evans & Whitelaw, 2009; Gibson, 1995; Syabbalo, 1998), this change is clinically meaningful. Likewise, in patients with the autoimmune disease Graves’ disease, Goswami et al. (2002) found that PImax increased from 69 to 81 cmH_2_O following the administration of carbiomazole (used to manage hyperthyroidism) over a 5 month period, and breathlessness improved. In both these examples, the increase in PImax was similar (12–13 cmH_2_O). Interestingly, this magnitude of change fell within the SDC of the present study. Given that the SDC was not reported by Keenan et al. (1995) or Goswami et al. (2002), it is not possible to ascertain with certainty whether these clinically meaningful changes reflect real changes in PImax or simply reflect the measurement error associated with their patient groups. Sadly, there is a lack of PImax and PEmax repeatability data in clinical populations. Consequently, determining the likely magnitude of the change in respiratory muscle strength (be that declining strength with disease progression or improvements following intervention) as part of the disease monitoring process is difficult to evaluate fully.

The LoA were initially developed to compare agreement between different measurement techniques (Weir, 2005), providing a range within which 95% of differences between two methods would lie (Bartlett & Frost, 2008; Bland & Altman, 1999). However, they can also be used to assess both repeatability and reproducibility (Bartlett & Frost, 2008; Bland & Altman, 1986; de Vet et al., 2006). Like the SEM, they have been used to assess the repeatability (Lomax & McConnell, 2009; Romer & McConnell, 2004; Silveira et al., 2021) and reproducibility (Silveira et al., 2021) of PImax.

Repeatability bias (i.e., difference) for PImax was generally excellent, being 2% at worst (Table 5) and only slightly larger for PEmax (4%, Table 6). This is similar to the ratio repeatability bias (3%) reported by others for PImax (Faghy & Brown, 2017; Lomax & McConnell, 2009; Romer & McConnell, 2004). Random error was always larger than bias for both PImax (Table 5) and PEmax (Table 6). This is to be expected given the multiple factors that impact random error, such as participant motivation and effort, mechanical variation of pressure meters, biological variability and chance factors.

Returning to the example of Rater_2_, the repeatability LoA of 0.98 ×/÷ 1.19 for PImax (Table 5) indicates that PImax will differ owing to measurement error by no more than 19% in either a positive or negative direction with repeat testing; for PEmax, this was 22% (Table 6). This means (assuming the bias is negligible) that participants with PImax and PEmax values of 89 and 111 cmH_2_O, respectively, will have values that differ owing to measurement error only by ±17 cmH_2_O for PImax (72–106 cmH_2_O) and by ±24 cmH_2_O for PEmax (87–135 cmH_2_O). From an analytical goals perspective, differences greater than these values would be required to be confident that any change observed with repeat testing was reflective of a real change and not simply measurement error. These values are not identical to those determined using the SEM or SDC.

Regardless of the statistic used, reproducibility was better than repeatability (except for LoA ratio bias, which was similar). Little has been reported on the impact of rater experience on recorded PImax and PEmax values. Only one study has examined ICC reproducibility in healthy adults measured by two different raters (Silveria et al., 2021). Although the reproducibility ICCs for PImax and PEmax were better in the present study than that reported by Silveria et al. (2021) (PImax, 0.98 vs. 0.91; PEmax, 0.97 vs. 0.84), those authors also observed better reproducibility than repeatability. It is likely that the natural day‐to‐day biological variation, likely subtle differences in testing conditions, and some characteristics of the participants (e.g., motivation, fatigue) account for the greater variability observed when testing over two separate days compared with a single testing session administered by different raters. Interestingly, PImax variability can be improved by administering an inspiratory muscle warm‐up. This has been shown to reduce the CV, increase the ICC and narrow the LoA (Lomax & McConnell, 2009).

Despite different testing experience, PImax was consistent between raters. Significant LoA ratio bias was observed between the rater with the most and least experience (Table 5), but the magnitude was still small. There was a trend for the rater with the greatest experience (Rater_2_) to have better ICC, smaller SEM, SDC and ratio random error values for PEmax. They were also able to elicit more consistent PEmax values on subsequent testing days (Table 1). However, these differences fell within normal levels (<10% or 10 cmH_2_O) of day‐to‐day variability (Black & Hyatt, 1969; McConnell, 2016).

Whether or not these small differences between raters have any practical meaningfulness depends on the analytical goal in question. Using the SDC as an example, if a change in PEmax of >41 cmH_2_O is anticipated (such as after expiratory muscle training), differences in rater experience would be of no consequence. However, if a change of ≤39 cmH_2_O is expected, rater experience will be important to be confident (95% level of confidence) that a real change had occurred; even so, a large degree of this change could simply reflect measurement error rather than a training response (Table 4). Given that past studies in healthy individuals have shown increases of 41–53 cmH_2_O in PEmax after expiratory muscle training (Griffiths & McConnell, 2007; Sapienza et al., 2002), differences in measurement error arising from rater experience would be of no practical significance.

PImax typically demonstrated better repeatability than PEmax (ICC, SEM, SDC and LoA ratio random error) and tended to be more reproducible (SEM, SDC and LoA ratio bias). This is likely to be an artefact of the flanged nature of the mouthpiece used. Theoretically, there is a greater propensity for air leakage during expiratory manoeuvres with a flanged mouthpiece, because pressure is positive during expiration, compromising the integrity of the lip seal. In contrast, the suction effect during inspiration helps to maintain a tight lip seal around the mouthpiece (Koulouris et al., 1998; Man et al., 2003; Troosters et al., 2005).

### 4.1 Limitations and additional considerations

Given that the present study focused only on young healthy adult males, the findings cannot be generalised to females, different age ranges or those with clinical conditions. Both PImax and PEmax are higher in adult males than in females (Black & Hyatt, 1969; Bruschi et al., 1992; Evans & Whitelaw, 2009; McConnell & Copestake, 1999; Reiter et al., 2006) and decline with age (Black & Hyatt, 1969; McConnell & Copestake, 1999; Reiter et al., 2006; Ringqvist, 1966). This relationship is non‐linear, with a greater slope evident after the age of 60 years (Evans & Whitelaw, 2009). However, respiratory mouth pressures are also impacted by physical fitness level, with this presenting a confounding factor especially when considering the impact of age (McConnell & Copestake, 1999). Other factors that have been shown to be correlated with respiratory mouth pressures include, height, body mass, body surface area and body mass index. These correlations are variable, demonstrating the complex interactions between physical characteristics and respiratory mouth pressure values (Bruschi et al., 1992; Evans & Whitelaw, 2009; McConnell & Copestake, 1999; Reiter et al., 2006).

Other sources of variability will come from PImax and PEmax manoeuvres. The volitional nature of the efforts necessitates not only coordination of the required musculature but also willingness and ability to exert maximal effort. This means that values will be impacted not only by participant motivation, but also by factors such as pain, breathlessness, thorax geometry, anxiety and comprehension of manoeuvre instructions (Aldrich & Spiro, 1995; Bruschi et al., 1992; Clanton & Diaz, 1995; Gibson, 1995: Syabbalo, 1998; Troosters et al., 2005). In the present study, participants were healthy and physically active, highly motivated and able to comprehend testing instructions. This might not always be the case (e.g., children, elderly, clinical groups), and respiratory mouth pressure measurements might be more variable as a result. It is possible that test administrator experience could be of greater importance in these situations.

Additionally, given that submaximal mouth pressure values can be equally consistent as maximal values, test administrators should take care to avoid confusing consistency with maximality (Aldrich & Spiro, 1995). A pressure–time trace of PImax and PEmax is useful in ensuring that a maximal sustained pressure is achieved (Evans & Whitelaw, 2009). This is not commonplace in physical activity studies where fit, healthy and motivated participants are recruited but is of greater importance in situations whereby the underestimation of PImax or PEmax will have ramifications for clinical decision‐making (Clanton & Diaz, 1995). In light of such factors, PImax and PEmax should not be used in isolation to identify respiratory muscle weakness. Instead, such tests can help to screen and track respiratory muscle weakness over time (Clanton & Diaz, 1995; Evans & Whitelaw, 2009; Gibson, 1995).

The type of mouthpiece and adequacy of the mouthpiece–face seal used during assessments should also not be overlooked. Flanged mouthpieces are associated with lower values than tube mouthpieces (Bruschi et al., 1992; Clanton & Diaz, 1995; Gibson, 1995; Troosters et al., 2005). The mouthpiece–face seal might also be compromised in those with facial weakness, leading to greater leakage, hence variability (Black & Hyatt, 1969; Syabbalo, 1998). The presence and size of a leak in the measuring equipment to prevent the cheeks and buccal muscles from contributing to pressure generation will also impact the values observed (Clanton & Diaz, 1995; Evans & Whitelaw, 2009; Gibson, 1995; Trooster et al., 2005). These factors all impact PImax and PEmax and should be taken into consideration when assessing their repeatability and reproducibility for any given participant group.

It is also important to acknowledge the significance of task learning with volitional measures of respiratory muscle strength. Multiple testing visits might be required before baseline PImax and PEmax can be established. Some studies have included three testing visits (e.g., Black & Hyatt, 1969; Maillard et al., 1998; Reiter et al., 2006), whereas others have included only two testing visits (Lomax & McConnell, 2009; Romer & McConnell, 2004; Silveira et al., 2021; Volianitis et al., 2001: Windisch et al., 2004) to account for task learning, with the possible addition of a standalone familiarisation visit. Given that our participants were permitted only one familiarisation visit before the two experimental visits, it is possible that a learning effect was present between experimental visits. However, given that no consistent increase in PImax or PEmax was observed between experimental visits, this is unlikely to have contributed to variability between testing days. This approach was chosen because multiple testing visits for establishing a baseline value are rarely permissible in clinical and occupational settings, and this is therefore more reflective of a real‐world scenario.

It should also be acknowledged that the sample size of the present study was small. A minimum sample size of 20 has been suggested when assessing measurement error, with a value closer to 50 being preferable (Atkinson & Nevill, 2001). Although the sample size of the present study is consistent with other studies in this area (Maillard et al., 1998; McConnell & Copestake, 1999; Romer & McConnell, 2004; Volianitis et al., 2001), it will have widened the CI for the LoA assessment.

Lastly, permitting individual raters to use their own words when instructing participants does introduce a source of variation. This approach was chosen because it reflects real‐world settings and gives test administrators verbal freedom when coaching participants and correcting errors. Our results do not suggest that this impacted reproducibility and repeatability in our group of participants. Nevertheless, following a prescribed script would standardise the measurement test procedure further, particularly when different test administrators are used.

## 5 CONCLUSION

It is concluded that the choice of statistics used to evaluate repeatability and reproducibility of PImax and PEmax should be given careful consideration, because they convey different information about the measured values, thereby impacting decision‐making in sports science and applied physiology settings. The data from the present study indicate that PImax and PEmax assessments can be administered in a repeatable and reproducible way if control measures are taken. The ICC will provide information on the relative consistency of PImax and PEmax values in a group of individuals but should be accompanied by a measure of test–retest variability if replicate values are of interest. The SEM and SDC are ideally suited for identifying the magnitude of measurement error (SEM) and, in turn, the change required in PImax and PEmax to indicate a real difference (SDC), whereas LoA are better suited for assessing agreement in PImax and PEmax between different raters or measurement tools.

Finally, reproducibility of PImax and PEmax was better than repeatability in our participant cohort of healthy young adult males. The repeatability and reproducibility of PImax was generally better than for PEmax, although the statistic chosen impacted the magnitude of the variation. If test administrators are trained to instruct in the techniques of mouth pressure assessments, the reproducibility of these values is acceptable, and rater experience does not pose a specific concern for replicate testing. However, whether the magnitude of measurement error is deemed acceptable should be based on the analytical goals in question. This might change depending on the participant group and the nature of any intervention designed to modify PImax or PEmax.

## AUTHOR CONTRIBUTIONS

All experiments were undertaken at the University of Portsmouth. Mitch Lomax, Josh Osofa, Nicola Armstrong, Michael Tipton and Gemma Milligan contributed to the conception of the work. Mitch Lomax and Josh Osofa undertook data acquisition. Mitch Lomax undertook data analysis and interpretation. Mitch Lomax, Josh Osofa, Nicola Armstrong, Michael Tipton and Gemma Milligan contributed to drafting of the work and revising it critically for important intellect content. All authors approved the final version of the manuscript and agree to be accountable for all aspects of the work in ensuring that questions related to the accuracy or integrity of any part of the work are appropriately investigated and resolved. All persons designated as authors qualify for authorship, and all those who qualify for authorship are listed.

## CONFLICT OF INTEREST

The research was conducted in the absence of any commercial or financial relationships that could be construed as a potential conflict of interest.

## Data Availability

The raw data that support the findings of this study are openly available in the University of Portsmouth data repository (Pure portal) at https://doi.og/10.17029/23937ab5‐1f54‐476c‐93cd‐6512df440619 under a CC BY 4.0 licence; file name ‘Respiratory mouth pressure_1065/MODERC/20’.

## References

[eph70348-bib-0001] Aldrich, T. K. , & Spiro, P. (1995). Maximal inspiratory pressure: Does reproducibility indicate full effort? Thorax, 50, 40–43. 7886647 10.1136/thx.50.1.40 PMC473703

[eph70348-bib-0002] Atkinson, G. , & Nevill, A. M. (1998). Statistical methods for assessing measurement error (reliability) in variables relevant to sports medicine. Sports Medicine, 26(4), 217–238. 9820922 10.2165/00007256-199826040-00002

[eph70348-bib-0003] Atkinson, G. , & Nevill, A. M. (2001). Selected issues in the design and analysis of sport performance research. Journal of Sports Sciences, 19(10), 811–827. 11561675 10.1080/026404101317015447

[eph70348-bib-0004] Bartlett, J. W. , & Frost, C. (2008). Reliability, repeatability and reproducibility: Analysis of measurement errors in continuous variables. Ultrasound Obstetrics & Gynaecology, 31(4), 466–475. 10.1002/uog.5256 18306169

[eph70348-bib-0005] Black, L. F. , & Hyatt, R. E. (1969). Maximal respiratory pressures: Normal values and relationship to age and sex. American Review of Respiratory Disease, 9, 696–702. 10.1164/arrd.1969.99.5.696 5772056

[eph70348-bib-0006] Bland, J. M. , & Altman, D. G. (1986). Statistical methods for assessing agreement between two methods of clinical measurement. Lancet, 327(8476), 307–331. 2868172

[eph70348-bib-0061] Bland, J. M. , & Altman, D. G. (1999). Measuring agreement in method comparison studies. Statistical Methods in Medical Research, 8, 135–160. 10501650 10.1177/096228029900800204

[eph70348-bib-0007] Boussana, A. , Galy, O. , Hue, O. , Matecki, S. , Varray, A. , Ramonatxo, M. , & Le Gallais, D. (2003). The effects of prior cycling and successive run on respiratory muscle performance in triathletes. International Journal of Sports Medicine, 24(1), 63–70. 12582954 10.1055/s-2003-37201

[eph70348-bib-0008] Brown, P. I. , Sharpe, G. R. , & Johnson, M. A. (2008). Inspiratory muscle training reduces blood lactate concentration during volitional hyperpnea. European Journal of Applied Physiology, 104(1), 111–117. 18560878 10.1007/s00421-008-0794-7

[eph70348-bib-0009] Brown, S. , & Kilding, A. E. (2011). Exercise‐induced inspiratory muscle fatigue during swimming: The effect of race distance. Journal of Strength and Conditioning Research, 25(5), 1204–1209. 20926968 10.1519/JSC.0b013e3181d67ab8

[eph70348-bib-0010] Bruschi, C. , Cerveri, I. , Zoia, M. C. , Fanfulla, F. , Fiorentini, M. , Casali, L. , Grassi, M. , & Grassi, C. (1992). Reference values of maximal respiratory mouth pressures: A population‐based study. American Review of Respiratory Disease, 146(3), 790–793. 1519865 10.1164/ajrccm/146.3.790

[eph70348-bib-0011] Clanton, T. L. , & Diaz, P. T. (1995). Clinical assessment of the respiratory muscles. Physical Therapy, 75(11), 983–995. 7480128 10.1093/ptj/75.11.983

[eph70348-bib-0012] de Vet, H. C. W. , Terwee, C. B. , Knol, D. L. , & Bouter, L. M. (2006). When to use agreement versus reliability measures. Journal of Clinical Epidemiology, 59(10), 1033–1039. 16980142 10.1016/j.jclinepi.2005.10.015

[eph70348-bib-0013] Eilasziw, M. , Young, S. L. , Woodbury, M. G. , & Fryday‐Field, K. (1994). Statistical methodology for the concurrent assessment of interrater and intrarater reliability: Using goniometric measurements as an example. Physical Therapy, 74(8), 777–788. 8047565 10.1093/ptj/74.8.777

[eph70348-bib-0014] Evans, J. A. , & Whitelaw, W. A. (2009). The assessment of maximal respiratory mouth pressures in adults. Respiratory Care, 54(10), 1348–1359. 19796415

[eph70348-bib-0015] Faghy, M. A. , & Brown, P. I. (2017). Whole‐body active warm‐up and inspiratory muscle warm‐up do not improve running performance when carrying thoracic loads. Applied Physiology, Nutrition and Metabolism, 42(8), 810–815. 10.1139/apnm-2016-0711 28288302

[eph70348-bib-0016] Fritz, C. O. , & Morris, P. E. (2012). Effect size estimates: Current use, calculations, and interpretations. Journal of Experimental Psychology, 14(1), 2–18. 10.1037/a0024338 21823805

[eph70348-bib-0017] Gibson, G. J. (1995). Measurement of respiratory muscle strength. Respiratory Medicine, 89(8), 529–535. 7480985 10.1016/0954-6111(95)90153-1

[eph70348-bib-0018] Gonzales, J. U. , & Scheuermann, B. W. (2006). Gender differences in the fatigability of the inspiratory muscles. Medicine & Science in Sports & Exercise, 38(3), 472–479. 16540834 10.1249/01.mss.0000189318.80061.fe

[eph70348-bib-0019] Goswami, R. , Guleria, R. , Gupta, A. K. , Gupta, N. , Marwaha, R. K. , Pande, J. N. , & Kochupillai, N. (2002). Prevalence of diaphragmatic muscle weakness and dyspnoea in Graves’ disease and their reversibility with carbimazole therapy. European Journal of Endocrinology, 147(3), 299–303. 12213666 10.1530/eje.0.1470299

[eph70348-bib-0020] Griffiths, L. A. , & McConnell, A. K. (2007). The influence of inspiratory and expiratory muscle training upon rowing performance. European Journal of Applied Physiology, 99(5), 457–466. 17186299 10.1007/s00421-006-0367-6

[eph70348-bib-0021] Hodges, P. W. , Gandevia, S. C. , & Richardson, C. A. (1997). Contractions of specific abdominal muscles in postural tasks are affected by respiratory manoeuvres. Journal of Applied Physiology, 83(3), 753–760. 9292460 10.1152/jappl.1997.83.3.753

[eph70348-bib-0022] Hopkins, W. G. , Marshall, S. W. , Batterham, A. M. , & Hanin, J. (2009). Progressive statistics for studies in sports medicine and exercise science. Medicine and Science in Sports and Exercise, 41(1), 3–12. 19092709 10.1249/MSS.0b013e31818cb278

[eph70348-bib-0023] Johnson, M. A. , Sharpe, G. R. , & Brown, P. I. (2007). Inspiratory muscle training improves cycling time‐trial performance and anaerobic work capacity but not critical power. European Journal of Applied Physiology, 101(6), 761–770. 17874123 10.1007/s00421-007-0551-3

[eph70348-bib-0024] Keenan, S. P. , Alexander, D. , Road, J. D. , Ryan, C. F. , Oger, J. , & Wilcox, P. G. (1995). Ventilatory muscle strength and endurance in myasthenia gravis. European Respiratory Journal, 8(7), 1130–1135. 7589397 10.1183/09031936.95.08071130

[eph70348-bib-0025] Khan, A. , Frazer‐Green, L. , Amin, R. , Wolfe, L. , Faulkner, G. , Casey, K. , Sharma, G. , Selim, B. , Zielinski, D. , Aboussouan, L. S. , Mckim, D. , & Gay, P. (2023). Respiratory management of patients with neuromuscular weakness. Chest, 164(2), 394–413. 36921894 10.1016/j.chest.2023.03.011

[eph70348-bib-0026] Koulouris, N. , Mulvey, D. A. , Laroche, C. M. , Green, M. , & Moxham, J. (1998). Comparison of two different mouthpieces for the measurement of PImax and PEmax in normal and weak subjects. European Respiratory Journal, 1(9), 863–867. 3229485

[eph70348-bib-0027] Laveneziana, P. , Albuquerque, A. , Aliverti, A. , Babb, T. , Barreiro, E. , Dres, M. , Dube, B. P. , Fauroux, B. , Gea, J. , Guenette, J. A. , Hudson, A. L. , Kabitz, H. J. , Laghi, F. , Langer, D. , Luo, Y. M. , Neder, J. A. , O'Donnell, D. , Polkey, M. I. , Rabinovich, R. A. , … Verges, S. (2019). ERS statement on respiratory muscle testing at rest and during exercise. European Respiratory Journal, 53(6), 1801214. 30956204 10.1183/13993003.01214-2018

[eph70348-bib-0028] LoMauro, A. , & Aliverti, A. (2019). Respiratory muscle activation and action during voluntary cough in healthy humans. Journal of Electromyography and Kinesiology, 49(6), 102359. 31568991 10.1016/j.jelekin.2019.102359

[eph70348-bib-0029] Lomax, M. , Iggleden, C. , Tourell, A. , Castles, S. , & Honey, J. (2012). Inspiratory muscle fatigue following race‐paced swimming is not restricted to the front crawl stroke. Journal of Strength and Conditioning Research, 26(10), 2729–2733. 22130403 10.1519/JSC.0b013e3182429af8

[eph70348-bib-0030] Lomax, M. , Kapus, J. , Wenn, S. , & Ušaj, A. (2019). The effect of inspiratory muscle fatigue on acid‐base status and performance during race‐paced middle‐distance swimming. Journal of Sports Sciences, 37(13), 1499–1505. 30724711 10.1080/02640414.2019.1574250

[eph70348-bib-0031] Lomax, M. , Massey, H. C. , & House, J. R. (2017). Inspiratory muscle training effects on cycling during acute hypoxic exposure. Aerospace Medicine and Human Performance, 88(6), 544–549. 28539142 10.3357/AMHP.4780.2017

[eph70348-bib-0032] Lomax, M. , & McConnell, A. K. (2009). Influence of prior activity (warm‐up) and inspiratory muscle training upon between‐ and within‐day reliability of maximal inspiratory mouth pressure. Respiration, 78(2), 197–202. 19342824 10.1159/000211229

[eph70348-bib-0033] Lomax, M. , Tasker, L. , & Bostanci, O. (2014). Inspiratory muscle fatigue affects latissimus dorsi but not pectoralis major activity during arms only front crawl sprinting. Journal of Strength and Conditioning Research, 28(8), 2262–2269. 24402450 10.1519/JSC.0000000000000379

[eph70348-bib-0034] Lomax, M. , Tasker, L. , & Bostanci, O. (2015). An electromyographic evaluation of dual role breathing and propulsion upper body muscles in response to front crawl swimming. Scandinavian Journal of Medicine in Science and Sports, 25(5), e472–e478. 10.1111/sms.12354 25640018

[eph70348-bib-0035] Maillard, J. O. , Burdet, L. , van Melle, G. , & Fitting, J. W. (1998). Reproducibility of twitch mouth pressure, sniff nasal inspiratory pressure, and maximal inspiratory pressure. European Respiratory Journal, 11(4), 901–905. 9623695 10.1183/09031936.98.11040901

[eph70348-bib-0036] Man, W. D. C. , Kyroussis, D. , Fleming, T. A. , Chetta, A. , Harraf, F. , Mustfa, N. , Rafferty, G. F. , Polkey, M. I. , & Moxham, J. (2003). Cough gastric pressure and maximum expiratory mouth pressure in humans. American Journal of Respiratory and Critical Care Medicine, 168(6), 714–717. 12857722 10.1164/rccm.200303-334BC

[eph70348-bib-0037] McConnell, A. K. (2016). Lung and respiratory muscle function. In E. M. Winters , A. M. Jones , R. C. R. Davison , P. D. Bromley , & T. H. Mercer (Eds.), Sport & exercise physiology testing guidelines (BASES) (pp. 63–75) vol 2*: Exercise & clinical testing*. Routledge.

[eph70348-bib-0038] McConnell, A. K. , Caine, M. P. , & Sharpe, G. R. (1997). Inspiratory muscle fatigue following running to volitional fatigue: The influence of baseline strength. International Journal of Sports Medicine, 18(3), 169–173. 9187969 10.1055/s-2007-972614

[eph70348-bib-0039] McConnell, A. K. , & Copestake, A. J. (1999). Maximum static pressures in healthy elderly men and women: Issues of reproducibility and interpretation. Respiration, 66(3), 251–258. 10364742 10.1159/000029386

[eph70348-bib-0040] Moravec, T. , Lomax, M. , Ušaj, A. , & Kapus, J. (2023). Inspiratory muscle fatigue at the swimming tumble turns: Its occurrence and effects on kinematic parameters of the turn. Frontiers in Physiology, 14, 1219520. 37383142 10.3389/fphys.2023.1219520 PMC10293647

[eph70348-bib-0041] Quan, H. , & Shih, W. J. (1996). Assessing reproducibility by the within‐subject coefficient of variation with random effects models. Biometrics, 52(4), 1195–1203. 8962450

[eph70348-bib-0042] Reiter, M. , Totzauer, A. , Werner, I. , Koessler, W. , Zwick, H. , & Wanke, T. (2006). Evaluation of inspiratory muscle function in a healthy Austrian population—practical aspects. Respiration, 73(5), 590–596. 16465046 10.1159/000091392

[eph70348-bib-0043] Ringqvist, T. (1966). The ventilatory capacity in healthy subjects an analysis of casual factors with special reference to the respiratory forces. The Scandinavian Journal of Clinical & Laboratory Investigations, 18, 1–179. 4283858

[eph70348-bib-0044] Romer, L. M. , & McConnell, A. K. (2004). Inter‐test reliability for non‐invasive measures of respiratory function in healthy humans. European Journal of Applied Physiology, 91(2–3), 167–176. 14605897 10.1007/s00421-003-0984-2

[eph70348-bib-0045] Santos, R. C. M. A. , Pinto, M. L. , Sant'Anna, C. C. , & Bernhoeft, M. (2011). Maximal respiratory pressures among adolescent swimmers. Revista Portuguesa de Pneumologia, 17(2), 66–70. 21477568 10.1016/s2173-5115(11)70016-1

[eph70348-bib-0046] Sapienza, C. M. , Davenport, P. W. , & Martin, A. D. (2002). Expiratory muscle training increases pressure support in high school band students. Journal of Voice, 16(4), 495–501. 12512637 10.1016/s0892-1997(02)00125-x

[eph70348-bib-0047] Silveira, B. M. F. , Pereira, M. C. B. , Cardoso, D. R. , Ribeiro‐Samora, G. A. , Martins, H. R. , & Parreira, V. F. (2021). New method for evaluating respiratory pressures: Concurrent validity, test‐retest, and inter‐rater reliability. Brazilian Journal of Physical Therapy, 25(6), 741–748. 34119441 10.1016/j.bjpt.2021.04.012 PMC8721068

[eph70348-bib-0048] Stratford, P. W. , & Goldsmith, C. H. (1997). Use of the standard error as a reliability index of interest: An applied example using elbow flexor strength data. Physical Therapy, 77(7), 745–750. 9225846 10.1093/ptj/77.7.745

[eph70348-bib-0049] Syabbalo, N. (1998). Assessment of respiratory muscle function and strength. Postgraduate Medical Journal, 74(870), 208–215. 9683973 10.1136/pgmj.74.870.208 PMC2360860

[eph70348-bib-0050] Terzano, C. , Ceccarelli, D. , Conti, V. , Graziani, E. , Ricci, A. , & Petroianni, A. (2008). Maximal respiratory static pressures in patients with different stages of COPD severity. Respiratory Research, 9(1), 8. 18208602 10.1186/1465-9921-9-8 PMC2244619

[eph70348-bib-0051] Troosters, T. , Gosselink, R. , & Decramer, M. (2005). Respiratory muscle assessment. European Respiratory Monograph, 31, 57–71.

[eph70348-bib-0052] Vaz, S. , Falkmer, T. , Passmore, A. E. , Parsons, R. , & Andreou, P. (2013). The case for using the repeatability coefficient when calculating test‐retest reliability. PLoS ONE, 8(9), e73990. 24040139 10.1371/journal.pone.0073990 PMC3767825

[eph70348-bib-0053] Verges, S. , Sager, Y. , Erni, C. , & Spengler, C. M. (2007). Expiratory muscle fatigue impairs exercise performance. European Journal of Applied Physiology, 101(2), 225–232. 17546459 10.1007/s00421-007-0491-y

[eph70348-bib-0054] Vincent, W. J. (2005). Statistics in kinesiology. (3rd ed.). Human Kinetics.

[eph70348-bib-0055] Volianitis, S. , McConnell, A. K. , & Jones, D. A. (2001). Assessment of maximum inspiratory pressure. Respiration, 68(1), 22–27. 11223726 10.1159/000050458

[eph70348-bib-0056] Volianitis, S. , McConnell, A. K. , Koutedakis, Y. , & Jones, D. A. (1999). The influence of prior activity upon inspiratory muscle strength in rowers and non‐rowers. International Journal of Sports Medicine, 20(8), 542–547. 10606219 10.1055/s-1999-9464

[eph70348-bib-0057] Volianitis, S. , McConnell, A. K. , Koutedakis, Y. , & Jones, D. A. (2001). Specific respiratory warm‐up improves rowing performance and exertional dyspnoea. Medicine and Science in Sports & Exercise, 33(7), 1189–1193. 11445767 10.1097/00005768-200107000-00017

[eph70348-bib-0058] Weir, J. P. (2005). Quantifying test‐retest reliability using the intraclass correlation coefficient and the SEM. Journal of Strength and Conditioning Research, 19(1), 231–240. 15705040 10.1519/15184.1

[eph70348-bib-0059] Wilson, S. H. , Cooke, N. T. , Edwards, R. H. T. , & Spiro, S. G. (1984). Predicted normal values for maximal respiratory pressures in caucasian adults and children. Thorax, 39(7), 535–538. 6463933 10.1136/thx.39.7.535 PMC459855

[eph70348-bib-0060] Windisch, W. , Hennings, E. , Sorichter, S. , Hamm, H. , & Criée, C. P. (2004). Peak or plateau maximal inspiratory mouth pressure: Which is best? European Respiratory Journal, 23(5), 708–713. 15176684 10.1183/09031936.04.00136104

